# Born digital or fossilised digitally? How born digital data systems continue the legacy of social violence towards LGBTQI + communities: a case study of experiences in the Republic of Ireland

**DOI:** 10.1007/s00146-021-01374-y

**Published:** 2022-02-08

**Authors:** Noeleen Donnelly, Larry Stapleton, Jennifer O’Mahoney

**Affiliations:** INSYTE Centre, Waterford Institute of Technology, Cork Road, Waterford, Ireland

**Keywords:** Digital data, Social media, Algorithms, AI, Culture, LGBTQI +, Gender

## Abstract

The AI and Society discourse has previously drawn attention to the ways that digital systems embody the values of the technology development community from which they emerge through the development and deployment process. Research shows how this effect leads to a particular treatment of gender in computer systems development, a treatment which lags far behind the rich understanding of gender that social studies scholarship reveals and people across society experience. Many people do not relate to the narrow binary gender options of male or female, and many people express their gender identity in much richer ways than the sex/gender binary female/woman and male/man Boolean terms will allow. We ask: are “born-digital” gendered datasets in digital systems experienced as marginalising by those who express their identity beyond the male/female binary? Case Study: Ireland. To answer this universal question, this paper presents the findings of an empirical case study of people in Ireland with diverse gender identities and expressions, and their experiences with public data systems and new technologies. In spite of great social changes in Ireland which have led to constitutional change in favour of LGBTQI + people, born-digital systems were experienced by respondents as embodying socio-cultural values which were no longer accepted in society at large. For many of the respondents, digital technologies routinely marginalise them in all kinds of ways. These systems keep alive violence and oppression long after civil rights have been enshrined in constitutional law. This study is just one example of the way assumptions about digital are disengaged from society-at-large. It is a call to arms to all who are passionate about socially-responsible technology.

## Introduction

The authors are lecturers in computer science and humanities with much involvement in diversity and inclusion within IFAC and the INSYTE research lab. The lead author is an LGBTQI + (lesbian, gay, bisexual, transgender, queer/questioning, intersex plus) activist and an industry professional with involvement in diversity and inclusion within the workplace. Through the lens of gender, this paper examines digital data collection systems used for everyday services like banking or applying for a driving licence, as well as social networking and new technologies such as predictive marketing and artificial intelligence. This paper focusses on the impact of these systems on the LGBTQI + community using a case study in Ireland. The case study was introduced in Ireland to explore the lived experiences and the perceptions of persons with technology in relation to their gender, where their gender identity and/or gender expression is outside of the sex/gender binary categories female/woman and male/man in Irish society. Gender identities, other than the binary identities of ‘male’ and ‘female’, are associated with queer culture under the acronym LGBTQ. LGBTQ represents a diverse group of sexualities including lesbian, gay and bisexual and a diverse group of gender identities, including transgender, under the umbrella term queer or questioning as there are many more sexualities and genders (Bolger and Killermann 2018). Transgender describes a person who has transitioned (or is transitioning) from living as one gender to another (Nestle et al. 2020; Feinberg 1998). Gender identities other than the binary gender identities of ‘male’ and ‘female’ include the gender identities transgender, genderqueer, genderfluid, gender non-conforming, passing and questioning (Nestle et al. 2020). Biological sex is a medical term used to express the sex assigned at birth. The classification of sex of an individual as female or male or intersex is derived from the characteristics of the individuals’ chromosomes, hormones and anatomy (Bolger and Killermann 2018). Cisgender is a term used to describe a person with a gender identity and a gender expression that matches their biological sex and have a gender that fits the societal norm (Agius and Tobler 2011; Bass et al. 2018; Bolger and Killermann 2018).

To date, there have been numerous studies within the LGBTQI + community in Ireland, and some of this research has included some data collection in relation to technology usage. However, the focus within these studies has been on sexual orientation—lesbian, gay, bisexual, other and mental health (Higgins et al. 2016) or sexual orientation and age (Higgins et al. 2011) or the transgender community and transphobia in Ireland (Haynes and Schweppe 2017). There has not been any research specifically focused on non-binary gender identities in Ireland and the experience with data collection systems and technology. However, within the transgender community research on transphobia in Ireland, data related to non-binary gender identities was collected (Haynes and Schweppe 2017) and the LGBTIreland Report (Higgins et al. 2016) research on mental health is to date the largest study of transgender people, and the first study with a sample of intersex people (Higgins et al. 2016, p 1). A mixed methods approach was utilised for this research to gain a broad view from the research group and also to gain an in depth understanding of the experiences of persons in the research group. In addition to this, a mixed methods approach for this research is in keeping with previous studies within the LGBTQI + community in Ireland (Donnelly 2011; Higgins et al. 2011).

At present, Irish legislation has come a long way over the past 28 years in terms of equality for LGBTQI + persons living in Ireland. Homosexuality was decriminalised in 1993, the Civil Partnership Act was passed in 2010, the same-sex marriage referendum was passed in May 2015 and the Gender Recognition Act was passed in July 2015 (Higgins et al. 2011). The ‘yes’ vote for same-sex marriage equality in 2015 by public vote, put Ireland on a world pedestal in terms of its advances in equality for the LGBTQI + community. It was a material expression of the enormous shift in the attitude of Irish society over the past 28 years towards those people who previously found themselves marginalised on the basis of their sexual orientation. In spite of this sea-change in Irish cultural attitudes towards LGBTQI + people since 1993, this paper uses empirical evidence to demonstrate that born-digital data sets continue to embody the marginalising heteronormative attitudes of the traditional Irish society which the civil rights movement sought successfully to change. This paper shows that the people in this case study continue to experience marginalisation as a result of the framing of born-digital datasets which capture data on gender. In particular, this paper focuses on the notion that these born-digital data systems, and the technologies which accompany them, reinforce gender discrimination through the design and the development of systems that marginalise and exclude genders other than the culturally constructed binary sex/gender categories of female/woman and male/man.

This paper proposes to answer: are “born-digital” gendered datasets in digital systems experienced as marginalising by those who express their identity beyond the male/female binary? Case Study: Ireland. The aims and objectives of this research are to examine the existing literature on gender and digital technology through the secondary research and to answer the following research questions through the primary research using a case study in Ireland:

(RQ1) For people within the LGBTQI + community:

- What is the diversity of gender identities and gender expression?
- What is the opinion on how and when gender data should be collected?

(RQ2) For people with diverse gender identities and gender expressions:

- What are their experiences with digital data collection systems?
- What are their experiences with social media?
- What are their experiences with new technologies?

## Literature review

The last section provided definitions of the key terms used in this research as well as introducing the general context and concepts of this research. This section will unpack the existing literature in the areas of: technology, culture and gender; digital data systems and gender and smart data and gender.

### Technology, culture and gender

Gender identity is a sense of self, a feeling of gender regardless of anatomy (Nestle et al. 2020; Butler 1999; Feinberg 1996). Gender expression is the outward style and behaviour in relation to the sense of self regardless of anatomy (Nestle et al. 2020; Butler 1999; Feinberg 1996). Persons born with what is labelled as, male sex characteristics, does not mean that persons must grow to be men or behave in masculine ways, these persons can be women and vice versa (Nestle et al. 2020; Butler 1999; Feinberg 1998). Some persons are comfortable with the cisgender sex/gender categories female/woman, male/man in society but this is not the case for everybody (Nestle et al. 2020; Butler 1999; Feinberg 1996, 1998). Gender, then, is heavily framed by social and cultural descriptors which frame expectations of how someone “should” look, behave, and speak, regardless of their individual gender identity. Culture shapes gender stereotypes via cultural values held by that given society. These values carry expectations about behaviours and characteristics (and relationships) deemed appropriate to women or men. In other words, gender functions to organise society based on the cultural meanings associated with being male or female (Schalkwyk 2000). The concepts of sex and gender binary norms and the direct mapping of sex to gender as natural and anything else is unnatural (Nestle et al. 2020) is “drilled into us through popular culture and education over years” (Feinberg 1998, p 3). Eckert and McConnell-Ginet (2013) suggest that there are no other easily obtainable ways of thinking about the identity of humans that are existing today and furthermore there will be an expectation “to pattern all kinds of things about ourselves as a function of that initial dichotomy” (Eckert and McConnell-Ginet 2013). Similar to how genderqueerness is described as the consequence of a “spectrum of gender” where “every spectrum turns out to be anchored by the same familiar two poles-male/female, man/woman, gay/straight” (Nestle et al. 2020 p 29). The systemic stereotyping of gender is echoed in technology, where technology, just as in all spheres of life, uses gender as an organising principle that replicates and maintains a gendered status quo. Consequently, categorising a persons’ gender into the cisgender sex/gender binary female/woman or male/man (Feinberg 1996) in digital data systems, without considering the diversity of gender identities and gender expressions, marginalises anybody outside of these restrictive binary gender categories.

“Born digital” refers to data-sets which starts its life in computerised data systems. Both technology and society coexist and technologies can influence human perception and human understanding of the world in addition to affecting what it means to be human (Ihde 1990). Technology and humans are not completely separate and unconnected entities, such that, technology is a tool that humans use to meet a goal (Tabachnick 2007). Technology is not a neutral object and the context and the conditions through which the technology is designed, influences the end product (Feenburg 2002; Noble 2018). Technologies designed under a technocratic model do not include the societal context of deployment. In contrast, technologies designed under a democratic model embrace the principle that technology must be designed for the context of the society for which it will be deployed and utilised (Feenburg 2002). The democratic model decentralises the hierarchy of control and power unlike the technocratic model where power relations, structure and regulate technology use, and design practices reinforce the values that shape how technology is designed (Feenburg 2002). Therefore, computerised data systems are shaped by socio-cultural forces in the design and development community and produce outcomes which embody the values and mindsets of the ICT development community from which they emerge. For example, body scanners used for security checks at airports are built on the notion that a person presenting as a woman will have ‘female’ body parts and a person presenting as a man will have ‘male’ body parts (TSA 2021). The advanced imaging technology (AIT) is used as a screening process such that “When you enter the imaging portal, the TSA officer presses a button designating a gender (male/female) based on how you present yourself. The machine has software that looks at the anatomy of men and women differently.” (TSA 2021).

There is also the notion that technology itself is gendered and computers are seen as a “male” domain in the west following the notion that men have “a natural affinity with technology” and “women supposedly fear or dislike it”. For instance, studies show how assumptions about girls perceived “incompetence” with technology use are informed by gendered social norms, impacting how girls view and interact with technology (Stepulevage 2001). In addition to gender, technology is also culturally constructed (Bray 2007). In today’s technoculture environment (Ihde 1990; Feenburg 2002), society’s patriarchal coupling of gender and technology “translates into everyday experiences of gender, historical narratives, employment practices, education, the design of new technologies, and the distribution of power across a global society in which technology is seen as the driving force of progress” (Bray 2007). The patriarchal cultural construct of technology as “masculine” is further highlighted by the fact that the workforce in western science, technology, engineering and mathematics industries is approximately 75% male (Daly et al. 2018, p. 2). Trauth (2013) shows that research on gender and digital systems from 1993 to 2013 focused on two main categories:

- %age women vs. %age men working in IT.
- Adoption and use of IT amongst men versus women.

Clearly, these impose a binary classification of gender so that “gender theorizing essentializes men as a single group and women as a single group” (Trauth 2013, p 284). There are serious implications of using a binary classification of gender in technology on people’s lives, contributing to a sense of marginalisation and exclusion for people who do not identify with binary classifications of gender. Ruberg and Ruelos (2020) maintain that dominant forms of binary demographic gender data do not account for the complexities that characterise many LGBTQI + lives. The authors highlight that gender and sexual identities are often in flux, resisting classification in fixed and discrete categories. However, there are clear attempts to progress technology past these limitations, into a postgender technoculture. Postgenderists argue that gender constitutes “an arbitrary and unnecessary limitation on human potential and foresee the elimination of involuntary biological and psychological gendering in the human species” through technology (Dvorsky and Hughes 2008, p 2). Theorists maintain that binary gender roles are restrictive, and their removal will facilitate more diverse self-expression and choice.

### Digital data systems and gender

Computers have a language known as machine language (Schmit 1995; Wakerly 2002). Machine language is a set of bit strings consisting of binary numbers, zeros and ones, that relate to instructions to allow for communication between humans, and digital machines and computers (Schmit 1995; Plant 1997; Wakerly 2002). Digital computers have a system program called an assembler (Wakerly 2002). The assembler software enables the direct translation of the symbolic assembly language of the software into machine language (Wakerly 2002). Thus, “all digital computers translate information into the zeros and ones of machine code, whether they are gathering information, telecommunicating, running washing machines, doing sums, or making videos” (Plant 1997). Plant (1997) depicts that, machines and computers are evaluated by their abilities to mimic humans. In addition to this, the criteria to make a machine, or programme a computer, to perform like a human, is normalised into the binary sex/gender identity categories of male/man and female/woman in society. There is evidence that these sex/gender norms and hierarchies have been historically enforced deeply within the birth of computerized systems forming the foundations for the transphobic algorithmic bias seen today (Hicks 2019). For example, Hicks (2019) shows that, prior to computerization of government systems in Britain, a British citizen could have their gender corrected on their government-issued identification (ID) cards to reflect their lived gender identity. However, since the digitisation of the government’s system, Britons attempting to correct their gender on their government-issued ID cards came up against the heteronormative gender binary digital data systems where this request was programmatically categorised and flagged as “a group of ‘exception’ cases that had to be dealt with as being aberrant from the norm” (Hicks 2019). Therefore, undoing these sex/gender stereotypes that are built into the criteria for programming the machine (Plant 1997) or the computer, neutralises gender. This concept echoes Haraway’s (1987) notion of the disembodied cyborg in a virtual world that has no gender identity.

### Social media, data and gender

Gender classification in digital data systems is also evident in the social media data made available from social networking platforms, such as Facebook, to advertising companies and researchers (Gilroy and Kashyap 2021). Social media technology companies collect data created by the end-users of their social networking sites within virtual communities and networks (Kaplan and Haenlein 2010). It is the social media technology companies, such as Facebook, who make the decisions on how to collect the data and how to make this data available (Bivens 2017; Gilroy and Kashyap 2021). This inferred information about us and our behaviour is then used as a reliable data source by advertising companies and coded as relationships between people, ideas and things into algorithms to predict what people like and want (Van Dijck 2013). Similar to the way in which the computerisation of government systems in Britain (Hicks 2019) made life difficult for people who did not fit into the program’s binary norms, these digital datasets reinforce marginalisation towards minority groups in society (Bivens 2017) despite the advances in human rights for minority groups such as the LGBTQI + community. Bivens (2017) highlights how Facebook present users with 58 options for gender identity but Facebook then translates these gender identities into three categories: women, men and non-binary, in accordance with an original software design decision. Furthermore “in the deep level of the database, non-binary users are reconfigured into a binary system.” (Bivens 2017). Facebook’s act of forcing the diversity of gender identities binary categories was intrinsic to the limitations of a recent quantitative analysis of digital data from Facebook’s advertising platform to show how sexuality is indicated socially (Gilroy and Kashyap 2021). The extent of these limitations where that it was not possible to differentiate between transgender and cisgender women and men nor was it possible to include non-binary people in the study and therefore this study was limited to a binary understanding of gender (Gilroy and Kashyap 2021) highlighting the marginalisation of genders outside of the heteronormative female/woman and male/man. Further to this, due to the intersectionality of gender and sexual orientation, Facebook’s data categorisation limited the ability of Gilroy and Kashyap’s (2021) research to account for the full spectrum of sexual identities and made sexualities, such as asexual, invisible.

### Smart technologies, data and gender

Gender identity and gender classification are also of particular interest to law enforcement for forensics, investigation and social justice and to commercial organizations for marketing and advertising and to others for social reasons (Alowibdi et al. 2013; Álvarez-Carmona et al. 2018). As a result, there have been numerous studies using data mining techniques to identify the gender of users on social media sites such as Twitter, YouTube and Flickr (Burger et al. 2011; Cheng et al. 2011; Peersman et al. 2011; Alowibdi et al. 2013). These studies have also utilised user generated content such as profile data, chat messages and comments from social networking sites like Twitter and YouTube, to build a catalogue of vocabulary to categorize text to predict the gender of the user. (Burger et al. 2011; Cheng et al. 2011; Peersman et al. 2011; Alowibdi et al. 2013). In addition to content based text mining, image based data from sites such as Flickr has been used with semantic data tagging of the images to predict the gender of the user (Eltaher and Lee 2015). “Experiments also indicate that function words, word-based features and structural features are significant gender discriminators” (Cheng et al. 2011). However, all these studies have defined gender identity as a binary classification problem consisting of two classes, ‘male’ and ‘female’ (Burger et al. 2011; Cheng et al. 2011; Peersman et al. 2011; Alowibdi et al. 2013; Eltaher and Lee 2015; Álvarez-Carmona et al. 2018). Shoshana Zuboff (2019) expresses the collection of online user data for translation into behavioural data for consumption by machine learning techniques and algorithms to predict our future behaviour as ‘surveillance capitalism’, “an information civilization shaped by surveillance capitalis and its new instrumentarian power will thrive at the expense of human nature and will threaten to cost us our humanity” (Zuboff 2019, p 11–12). Surveillance processes involve the social sorting of people into categories which are based on statistical averages or norms (Ball et al. 2009; Kafer and Grinberg 2019). The act of normalisation on society, generates discrimination, exclusion and marginalisation towards anyone who does not fit into the predefined societal norms of surveillance processes, especially with categories relating to gender and sexuality (Ball et al. 2009; Kafer and Grinberg 2019). Feenburg (2002) maintains that it is the “antidemocratic values that govern technological development” rather than the technology itself that have a negative impact on society and the computer systems behind the face of social media and artificial intelligence are not impartial to biases and discrimination, they are programmed by humans who make design decisions using their own judgement and values, which in some cases may be sexist, racist or unjust (Noble 2018). Furthermore, there is no policy or governance for professional ethics in artificial intelligence (AI), encompassing machine learning techniques, algorithms, automated decision making systems and robotics, in Europe (Madiega 2019). Although machine learning is about the flexibility of the computer to reprogram itself without human intervention, the foundational algorithms on which AI technology is built utilises hardcoded data biases such as the gender binaries of male and female which are the object of this study. These deep-rooted biases and the lack of an ethical component in digital data systems and smart data technologies are programmed by humans. The necessity of changing how gender is encoded and interpreted in data are paramount, “especially in the era of algorithms and big data when the issue of who is or is not ‘counted’ profoundly affects visibility, access, and power in the digital realm (Ruberg and Ruelos 2020, p 1). If LGBTQI + people are not “counted” they are further marginalised and underrepresented in any outputs generated from big data. The mainstream cultural values which gave rise to and maintain a binary view of gender will continue to be perpetuated in the design of discriminatory data systems, and the systems, in turn, will shape our cultural values, prolonging a cycle of marginalisation. This research explores the experiences of digital data systems, social media and new smart technologies among gender diverse persons within the LGBTQI + community in Ireland. This research sheds light on a more general problem which goes beyond gender, to deep, under-the-surface biases in digital data systems and control and automation technology development which remain poorly understood.

## Research design

The mixed methods design for this research utilises a questionnaire for the quantitative methods and a focus group discussion for the qualitative methods. The data collection phase and the data analysis phase for each of the quantitative and qualitative methods were executed concurrently. Following from the data collection phase and the data analysis phase, the two sets of results are merged together into the interpretation of the findings.

### Inclusion criteria

This survey was conducted amongst persons in the LGBTQI + community in Ireland and amongst persons who have a gender identity and/or a gender expression that goes beyond the gender binary of simply ‘male’ or ‘female’ or ‘man’ or ‘woman’ in society.

### Data collection methods: survey design

Firstly, the survey for this research was designed by researching the existing surveys within the LGBTQI + community. The existing surveys include the Travel and tourism for lesbians in Ireland survey (Donnelly 2011), the Visible Lives survey (Higgins et al. 2011), the Trans PULSE survey (Trans PULSE Project Canada 2012), the LGBTIreland survey (Higgins et al. 2016) and the STAD: Stop Transphobia and Discrimination Report: 2014–2016 (Haynes and Schweppe 2017). Following from this research, a list of options was formed for the question on gender identity and the question on sexual orientation. All other questions were specifically designed for this research. Secondly, the survey was designed with a mixture of closed questions and open questions and all selection list questions included an open selection box for other answers. The profiling data on the survey includes gender identity, gender expression and sexual orientation. Age and location were also collected in case they were required for testing for relationships between answers. However, they were optional fields.

*Gender Identity:* the data related to the gender identity of the respondents was collected on the survey using a mandatory question containing a list of options where multiple selections could be made. There was also an option to include a more appropriate gender identity that was not available on the list provided.

*Sexual Orientation:* the data related to sexual orientation was collected on the survey using a mandatory question containing a list of options where one selection could be made. There was also an option to include a more appropriate sexual orientation than provided on the list.

*Sample Size*: Table 1 below highlights the sample size for this research in comparison to the sample size in the following surveys: the Visible Lives survey (Higgins et al. 2011), the LGBTIreland survey (Higgins et al. 2016) and the STAD: Stop Transphobia and Discrimination Report: 2014–2016 (Haynes and Schweppe 2017). This research is closely aligned with the sample sizes from the STAD: Stop Transphobia and Discrimination Report: 2014–2016 (Haynes and Schweppe 2017). There was a smaller percentage of respondents with a gender identity of *unsure* in this research, however, there was a larger percentage of respondents with non-binary genders and other genders in this research compared to the STAD Report (Haynes and Schweppe 2017). This research had a smaller percentage of respondents with a transgender identity and a much larger percentage of respondents identifying as bisexual.

**Table 1:** Sample size—gender identity

Research/Gender identity	Visible Lives Sample (Higgins et al. 2011)	The LGBTIreland Sample (Higgins et al. 2016)	STAD Sample (TENI, Haynes and Schweppe 2017)	Survey Sample
Lesbian/gay female				
Female	27.3%	26.5%	n/a	^a^
Gay male				
Male	65.0%	38.6%	n/a	^a^
Constant and clear identity as a woman	n/a	n/a	37.0%	50.0%
Constant and clear identity as a man	n/a	n/a	25.0%	20.7%
Unsure	n/a	n/a	13.0%	5.2%
Variable or fluid non-binary gender identity	n/a	n/a	13.0%	13.8%
Constant and clear non-binary gender identity	n/a	n/a	7.0%	10.3%
No gender identity	n/a	n/a	2.0%	1.7%
Transgender	7.0%	12.3%	^b^	5.2%
Intersex	n/a	2.0%	n/a	1.7%
Cisgender	n/a	n/a	n/a	20.7%
Other Identity	0.7%	6.3%	^b^	5.2%
Bisexual	9.00%	14.40%	n/a	29.3%
Total	144	2264	164	58

^a^The Visible Lives survey and the LGBTIreland survey presented their figures for binary genders male and female coupled with sexual orientation. The purpose of this research is to decouple sexual orientation and gender identity and to go beyond the binary options. For example, someone can be gay or lesbian and not identify with the genders male or female. Table 2 below highlights the sample size by sexual orientation within this research

^b^The STAD survey was conducted within the transgender community and the identity transgender was collected under a question relating to transgender identities rather than gender identity

**Table 2:** Sample size—sexual orientation

Sexual orientation independent of gender	Lesbian	Gay	Bisexual	Queer	Heterosexual	Other
This Research	29.3%	20.7%^c^	29.3%	8.6%	8.6%	3.4%

^c^5% identify as female

*Advertising and recruitment for survey*: in the current COVID-19 pandemic, a link to the survey questionnaire was distributed online by contacting various known LGBTQI + communities to disperse or advertise a link to the survey among the LGBTQI + community in Ireland. In addition to this, a link to the survey was shared among the researcher’s friends and colleagues within the LGBTQI + community in Ireland.

### Data collection methods: focus group

Within the qualitative research component of this research, a focus group of four participants was conducted. The participants were recruited via the last survey question that gave the researchers contact details or an option to leave a number if they would like to participate in the focus group discussion. This approach enabled the validation of the participant against the survey results given. Due to the COVID-19 pandemic, the focus group was conducted online using a private and secure video conferencing room via Zoom technology. All participants were contacted before the focus group to ensure that they had WIFI, a device—laptop/tablet/mobile phone and zoom software and that they were familiar with zoom. The meeting duration was one hour and the questions were taken from the open questions in the survey to explore the experiences of persons with technology and data collection systems in relation to their gender identity. The meeting was recorded in zoom and the participants gave their consent to the recording. All participants were asked to keep the focus group discussion confidential and between ourselves.

*Gender Identity:* within the focus group, the participants expressed their gender identity through the focus group discussion rather than being asked the question directly to avoid the assumption that everybody identifies with a gender. Also note that, observational gender related data does not apply to this study due to the nature of gender as a social construct and the fact that the perception of the gender of a person is different for everybody based on experience, knowledge and self-identification and self-expression.

*Age and Location:* These data were not collected during the focus group discussion to protect the anonymity of the focus group participants.

### Ethical issues

Due to the sensitivity of the research topic and the fact that this research is dealing with people in addition to facts and figures, ethics are an important part of this research. In the current COVID-19 pandemic, a link to the survey questionnaire was distributed online by contacting various known LGBTQI + communities to disperse or advertise a link to the survey among the LGBTQI + community in Ireland. With regard to the focus group, the researcher asked for consent from the participants to record the discussion and the researcher informed the participants when the record button was turned on. In addition to this, the focus group participants were given aliases within the transcription of the audio file and care was taken in writing findings so as the participants cannot be identified by any of the details. The participants in both the focus group and the questionnaire survey, were guaranteed confidentiality of the data collected and the option of remaining anonymous and all participants and respondents in this research were made aware of what the research is for and how the research will be utilized.

## Findings and discussion

This section highlights the key findings from this case study in Ireland to highlight: the diversity of gender identities and gender expression; the opinions on how and when gender data should be collected; experiences with digital data collection systems; experiences with new technologies; experiences with social media.

### Gender identity and gender expression

There were 18 unique gender identities selected by the respondents. 26% (15) of the respondents selected one, a combination of, or a combination including the following non-binary gender identities: Constant and clear non-binary gender identity; Variable or fluid non-binary gender identity; No gender identity; Unsure; Intersex; Other: Epicene; Other: Queer. 3 of these respondents also selected a constant and clear gender identity as a woman and also 1 selected a constant and clear gender identity as a man. 5% (3) of the respondents selected transgender, 2 also selected a constant and clear gender identity as a man and 1 respondent also selected other: Woman but with a pinch of queerfloatiness. 9% (5) of the respondents selected cisgender only and 60% (35) of respondents selected a constant and clear binary identity as either a man or a woman and some of these respondents also selected cisgender. These results show that gender identity goes beyond one of two binary categories of gender.

The data related to gender expression was collected on the survey using a mandatory question containing three scales where a selection on each was required. One scale was for feminine expression, one scale was for masculine expression and one was for androgynous expression and each of the three scales had points with various intervals ranging from 0 to 100%. There were 34 unique combinations of gender expression found. Only 5% (3) respondents indicated that their gender expression was 0% androgynous and, 100% feminine/0% masculine or 100% masculine/0% feminine. These respondents also indicated that they relate to a constant and clear gender identity as either a woman or a man and they are either gay or bisexual.

These results are reflective of the existing literature highlighting that there is not always a direct mapping from the binary female or male sex assignment at birth to gender binaries of woman or man in society. These findings support the assertions of Nestle et al. (2020) and Feinberg’s (1996, 1998) gender research. These findings show that, even if a person identifies with a binary gender identity as a man or as a woman or with a non-binary gender, there are many ways in such persons express their gender and there is an array of language that is used to describe the inner sense of gender and being. The results also highlight that there is not only one representation for what a woman looks like or how a woman behaves nor is there only one representation for what a man looks like or how a man behaves. This is evident from the array of feminine, masculine and androgynous combinations of gender expression across the respondents who also identify as a woman or a man. Misgendering is a theme that came across in this research. Misgendering refers to when a person identifies as one of the binary genders and is mistaken by society for the opposite gender. The concept of misgendering highlights the public’s automatic binary sorting of persons into man and woman based on gender expression, even if it is unconscious.

The following sections explore the experiences of this group of people when they encounter digital data systems, new AI and smart technologies and social media where they are forced to choose a binary gender option in order to proceed with the digitalized interaction.

### Experience with digital data systems

**Fig. 1:**
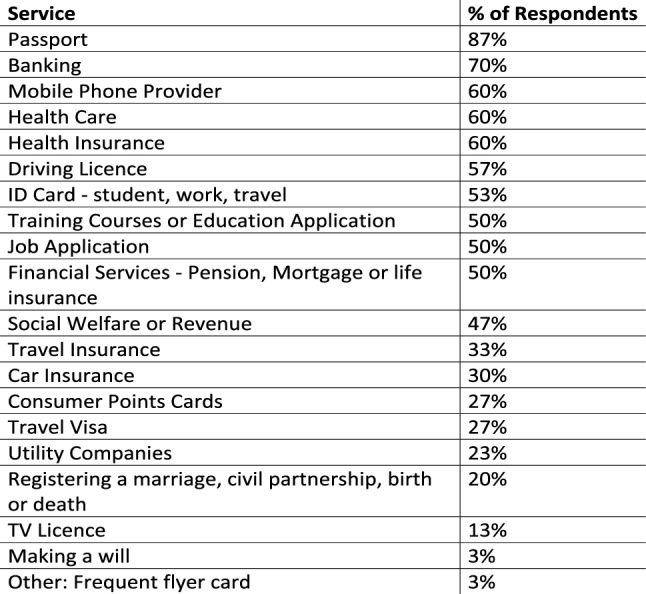
Frequency of services forcing a gender binary option

Figure 1 presents the services 31 respondents selected as services through which they experienced or encountered data collection forms where they felt forced to choose a gender with only the binary options of ‘male’ or ‘female’. Almost a quarter (23%) themselves selected one of the non-binary gender identities provided, 9% identified as a man or a woman and transgender and 69% identified with a binary gender. Respondents were asked if any of the service providers listed in Fig. 1 enabled users to update records to reflect a correct gender. Only 1 respondent indicated that they had successfully updated their gender and another respondent indicated that most services they used did enable updates to gender related data but that they found it was difficult to enact these changes in practise. Both respondents identified as transgender. Survey Respondent ID #46 explained:

“I am grateful that I have been able to change my gender relatively easily with most services due to the existence of the gender recognition act, however this does not extend to nonbinary people in Ireland and therefore those same services who would correct my gender with relative ease would likely refuse a nonbinary person.”

93% of people did not feel a need to ask for gender information to be changed, or had not asked for changes. Of these, over two thirds (68%) provided the following reasons:

1. don’t need to;
2. I am female;
3. I’m not personally impacted, but know it affects others.
4. It feels like a personal invasion;
5. There’s no point or I am not bothered;
6. I’m a gay man and have no reason.

5 respondents identifying with a non-binary gender, indicated that it is “exhausting” to get gender data updated and some indicated discomfort asking for the updates. Survey Respondent ID #66 explained:

“I would constantly be asking and it would be too time-consuming. It would take too much of a fight. I don't have the time and energy to face the discrimination I expect I would experience. I would have to come out publicly as gender fluid.”

One of the focus group participants, Mary, gave a detailed account of the time that she went for an interview for a housing application in Ireland in comparison to when she went to open a post office account in Ireland. Mary identifies as a woman and Mary is very happy to fill out forms and tick female. Mary is transgender and has gender recognition. The overall experience with the application for housing was a positive one for Mary but this really came down to the person whom Mary was dealing with. The digital data system in use by this government social department allowed Mary’s binary gender of ‘female’ to be input but then when Mary also selected ‘yes’ to having kids, the system asked about Mary’s husband. Another example supporting the findings in the research of Hicks (2019) and Ruberg and Ruelos (2020), that digital data systems reinforce heteronormative relationships built on gender stereotyping even though LGBTQI + rights have advanced. This question forced Mary into a situation where she had to explain her life story. However, in this instance, Mary explained that it was an okay experience because “I think sometimes the person you meet, you can be lucky or unlucky … she was so interested in and intrigued and so friendly and so understanding not to invade my privacy and only wanted me to answer questions that I was comfortable with.”. 2 focus group participants described that it is upsetting for non-binary persons to provide gender data on forms. Mary explained that they must either fill them out or explain themselves and give far more information than is required. Having to repeatedly explain herself as a result of the application forms requiring proof of gender identity was upsetting: They don’t just take “my word for it—I am a woman sitting in front of you’. 3 focus group participants asked “what do they need the gender data for anyway?”. This question was also echoed in the survey results. Over half (53%) of all survey respondents gave their opinion on how gender related data should be asked for on application forms. 18% felt that all genders should be available to select, 23% wished that an open text box be provided, and one of these respondents also opted for a list of all genders. Some questioned if it should be collected at all or that it should be optional. 18% felt strongly that it should be gathered only when clearly essential.

Another focus group participant Sarah described working on a recent project, within the life insurance industry, where the topic of non-gender binary came up. Sarah recounts the discussion between IT and the claims department in which the customer facing employees work: “… This is a massive thing that we try to get this absolutely right, that people are getting the right gender right and their title … we looked at all the different options with male, female, other, undefined, non binary”. However, IT discovered that the front end system that the claims department were using could be updated to take data from the client but there was a problem with the downstream legacy systems. In other words, the customer facing input system could be updated to look inclusive of non-binary genders but these inputs are eventual translated into binary gender options within the legacy system. This insight from the focus groups discussion also supports Hick’s (2019) findings.

Our evidence shows that, since the Irish Gender Recognition Act in 2015, most services in Ireland accommodate binary gender updates to system records for trans-binary persons with gender recognition. However, in spite of these constitutional changes which advanced the rights of LGBTQI + people, in Ireland today transgender persons without gender recognition, non-binary persons and non-gendered persons are marginalised and forced to explain themselves when they are trying to access the most basic automatic online services such as applying for a driving licence or taking out a post office account. These services are only accessible following provision of mandatory gender data with only two options ‘Male’ or ‘Female’. If a different gender is marked on the application outside of the M/F boxes, the application will not be accepted. Some respondents described the experiences as very upsetting, exhausting and marginalising. These people feel pushed into vulnerable interrogating situations as a result of the design of the automated data collection system.

The next section looks at the findings in relation the experiences of the survey respondents and the focus group participants in relation to new AI and smart technologies.

### Experience with technology: AI, smart data and marginalisation

**Fig. 2:**
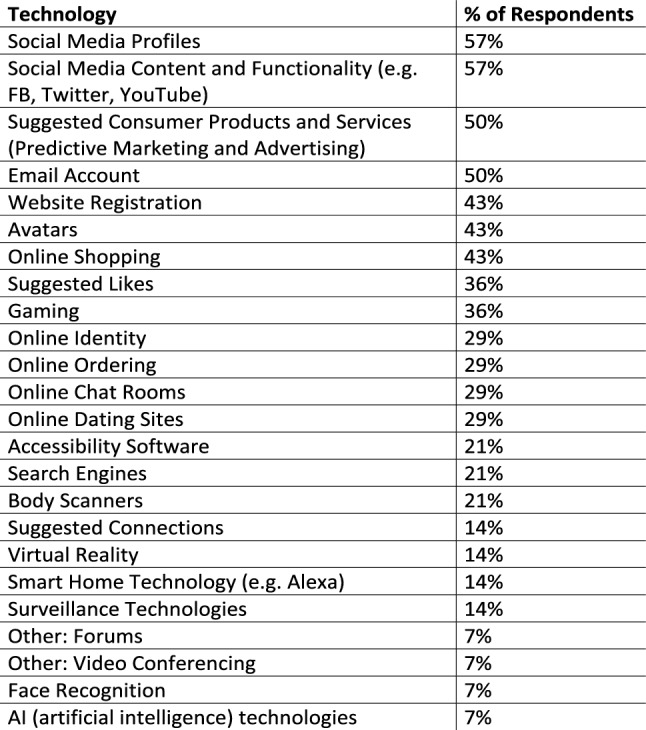
Frequency of technologies with a negative experience

50% of the respondents filled out the survey section on experience with technology. 48% of these respondents indicated that they encountered issues with technology in relation to their gender identity or their gender expression. Figure 2 below highlights the frequency of technologies indicated as the respondent having a negative experience with them. Social media profiles, social media content and functionality, predictive marketing, email accounts, website registration, avatars, online shopping, suggested likes, gaming, online Identity, online ordering, online chat rooms/forums and online dating sites are technologies that participants in this research experienced negatively in relation to their gender identity or their gender expression. In addition, accessibility software, search engines, body scanners, suggested connections, virtual reality, smart home technology like google assist, surveillance technologies, video conferencing, face recognition and AI (artificial intelligence) technologies are also reported as problematic.

All persons indicating part of their gender identity as being transgender, also indicated that they are having negative experiences with “social media profiles” and “social media content and functionality”. The frequency of negative experiences with “social media profiles”, “social media content” and “functionality” and “online ordering” was greater for persons indicating part of their gender identity as transgender than not. Respondents indicating all or part of their gender identity as transgender or non-binary had a similar frequency of negative experiences with “website registration” and “online shopping” compared to respondents identifying with only a binary gender, “woman” or “man”. Also, none of the respondents identifying with only a binary gender, “woman” or “man”, reported negative experiences with “online chat rooms”. When asked, 55% of respondents indicated that they agree with the statement “technology is gender biased” they reported an average score of almost 8/10 where 10 is totally agree and zero is do not agree. On the other hand, 14% felt neutral about this statement and 31% strongly disagreed with the statement with an average rating of 1.2. Whilst the overwhelming experience for 55% of the group was that these technologies are fundamentally cisgender male dominant, there was internal disagreement. In addition to the survey respondents, all focus group participants agreed that technology is gender biased. Cillian explains why they think that technology in biased.

“Ireland is every bit as patriarchal as the U.S. or anywhere else … and it just seems in technology development, there seems to be a sense of cowboy culture … among code … it just seems to be overwhelmingly male or cis gender male”

**Fig. 3:**
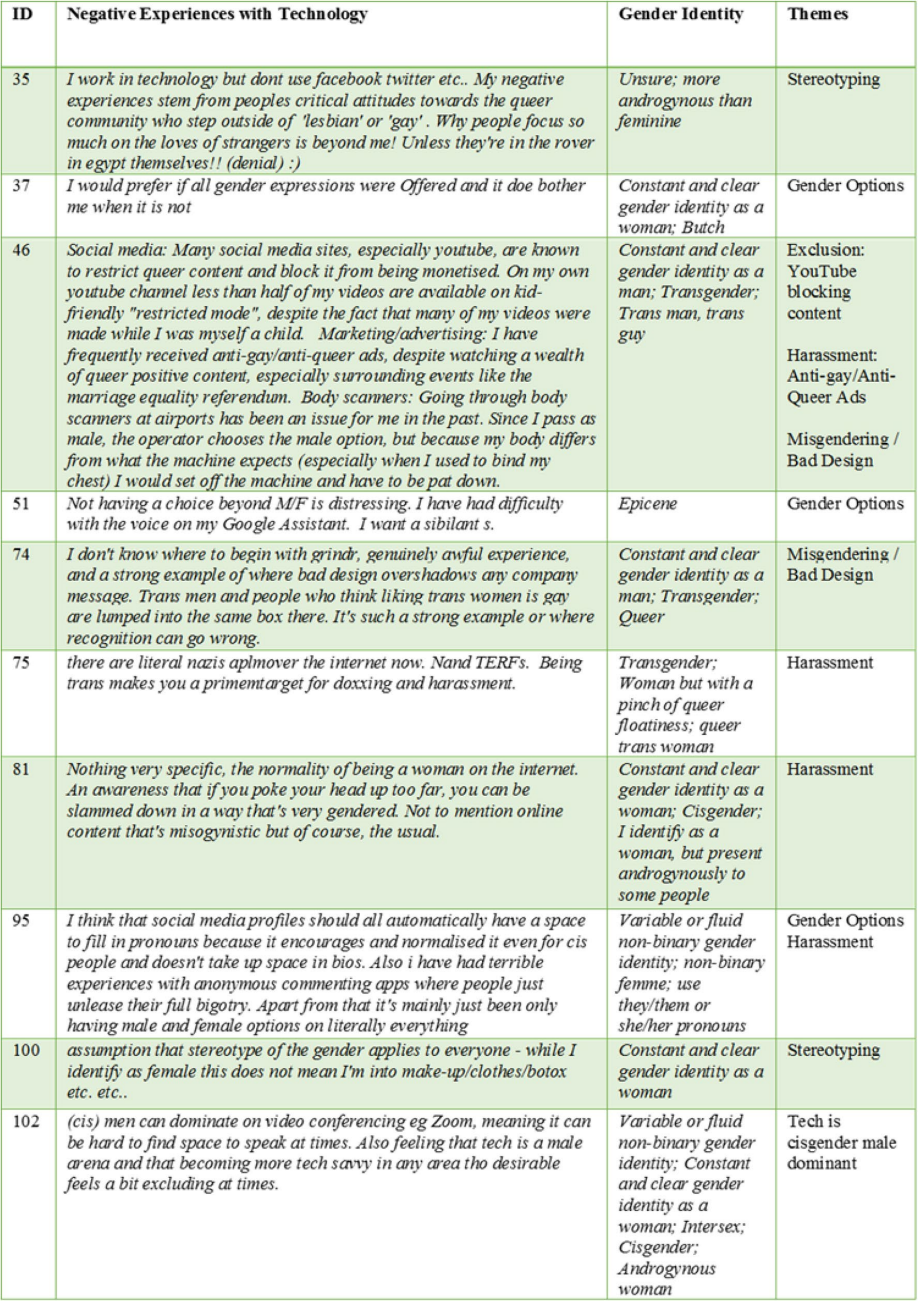
Comments about negative experiences

10 respondents left a comment about their negative experiences with technology and online socialising, highlighted in Fig. 3 below. Major themes included online harassment, misgendering, exclusion and the need for more gender options or a free form field for gender. They described how online automation technologies were badly designed because they stereotyped them and embodied stereotypical, incorrect assumptions. For example, systems made assumptions about who and what services or products they would like based on an assumed gender as well as black listing content.

#### Experience with predictive marketing and advertising

The findings in Fig. 2 show that predictive marketing was ranked second to social media, as a technology that persons in this research experienced negatively. Predictive marketing techniques involve collecting data and translating it into behavioural data for consumption by machine learning techniques and algorithms to predict our future behaviour. This process is referred to as ‘surveillance capitalism’ (Zuboff 2019, p 11–12) in the literature review. It is evident from the findings in Sect. 4.3 that, the algorithms behind predictive marketing and advertising make assumptions based on the sex/gender binary categories female/woman and male/man. There is evidence in the findings that if a person identifies as female, the person is categorised as being interested in makeup, clothes and botox. However, it is clear from this research that clothing, makeup, interests, hairstyles and much more are all attributes of gender expression which can be measured on a scale of femininity, masculinity and androgyny. There are many permutations of femininity, masculinity and androgyny and they could change day to day as well. In fact, this research showed, in Sect. 4.1, how diverse gender expression is, even among persons who identify with a binary gender of woman or man. Therefore, predictive marketing tools are based on stereotyping gender into two categories and if a person does do not fit into one of these two categories that person is marginalised even further for being different from the expected norm. Another respondent experienced anti-gay-queer ads even though he had watched an abundance of content in relation to the same-sex marriage referendum.

#### Other experiences with technology

The findings in Figs. 2 and 3 also give evidence to negative experiences with body scanners. Body scanners in airports are programmed to identify one of two body categories, male or female (TSA 2021). However, the body scanner needs the gender to be input into the machine before the person walks through and this gender identifier is based on the officer’s interpretation of gender expression. Therefore, if the person is a masculine female passing as a man, the body scanner will detect an anomaly and the person will have to be pat down. Yet another example of an invasive technology with extremely inhuman consequences for the non-binary person. All of this hassle and interrogation because the technology is based on the narrow assumptions of a sex/gender binary and the interpretation of what a man and a woman look like. In addition to this, there were a few other findings: there are gender binary assumptions made about persons once they are over a certain age and these assumptions are coded into systems; cisgender men can dominate on video conferencing technology such as Zoom; all gender expressions should be offered online where gender is required. It is distressing not having a choice beyond male or female. Lastly, the comments about the negative experiences of the participants in this research, highlight that people in marginalised groups in society feel that there are patriarchal technological structures in play.

### Experience with online socialising

**Fig. 4:**
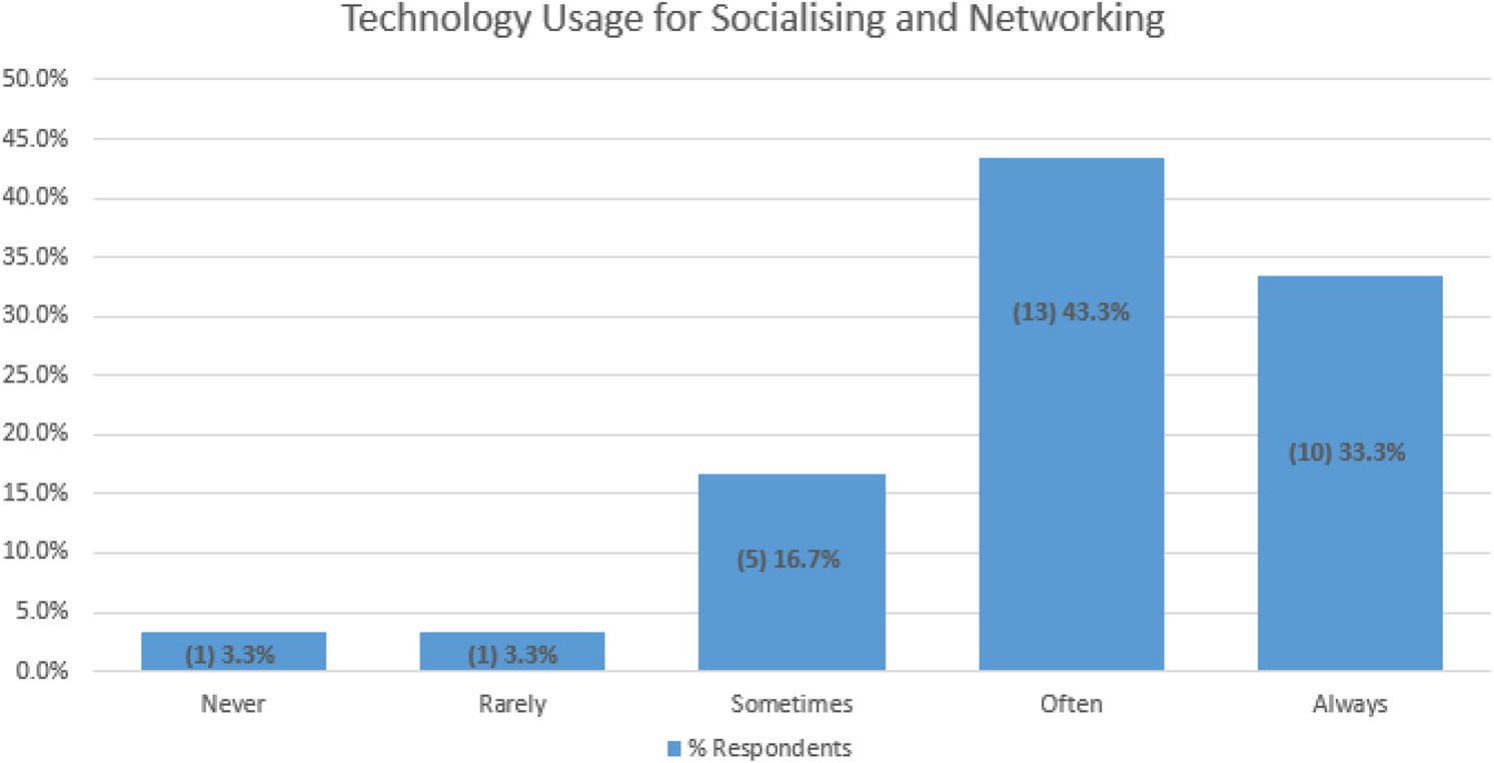
How often do you use technology (go online) for socialising and networking?

The findings in Fig. 2 above, show that social media profiles and social media content and functionality (FB, Twitter, You Tube) were ranked as the number one technology that persons in this research experienced negatively. Figure 4 below highlights the frequency of usage for online socialising and networking across 50% of the respondents.

These survey respondents were also asked about their experience with socialising online. The answers to “Online social spaces are inclusive of all genders” and “Online social spaces are inclusive of LGBTQI + people” are closely aligned. The respondents who “rarely” go online to socialise answered “unsure” to all of the question related to their experience socialising online. All respondents indicating a gender identity as a man or a woman and also transgender, answered “strongly agree to you have experienced gender discrimination attacks in online social spaces”. In addition to this, all respondents indicating a gender identity as a man or a woman and also transgender, agreed that online social spaces enabled them to express their gender, however, they answered “disagree” or “unsure” to the questions about online social spaces being inclusive of all genders and LGBTQI + people, you can be you in online social spaces and you feel safe in online social spaces. A significant statistical difference was found between respondents indicating a binary gender identity and all other respondents in relation to the answer to “Online social spaces enable you to express your gender identity”. The focus group participants expressed that during the Covid19 public socialising restrictions and lock downs, social media and video conferencing technologies have enabled people to socialise and connect in a virtual world. In addition to this, because events and meet ups have gone virtual, location is not a factor. For example, Cillian is able to join weekly coffee meetups that are usually located about 120 km from home. This was not possible when it was in a public space and not held virtually. In addition to this, Ali can attend global events that were not feasible to travel to when they were not held virtually. Socialising technology was invaluable for them during the lockdown. However, even though there are some advantages Mary prefers to meet in public rather than online so as she can confirm in person herself who see is meeting with. Mary doesn’t mind doing zoom calls but she’s not fond of them and she’s not fond of sharing details on it. The reasons are safety and protection. Mary explains:

“I prefer public. I don't feel safe on social media … I just think it's this for my protection. That's the way I feel.”

These findings highlight that, online socialising is frequently used by the participants in this research and there are mixed views about the gender inclusiveness and the LGBTQI + inclusiveness of online social spaces. Most persons identifying with a non-binary gender identity do not agree that, or are unsure if, online social spaces are inclusive of all genders and LGBTQI + people. In addition to this, persons identifying with a non-binary gender identity do not believe that online social spaces enable them to express their gender identity in comparison to persons identifying with binary genders including trans-binary. All participants in this research indicating a trans-binary gender identity, agreed that online social spaces enable them to express their gender. However, they are either unsure or don’t feel safe to express their gender identity in social spaces and they don’t agree that social spaces are inclusive of all genders and LGBTQI + persons or else they are unsure if these spaces are inclusive. It is evident that trans-binary persons feel this way about online social spaces because they have all experienced gender discrimination attacks in online social spaces. Further to this, harassment and discrimination are prevailing themes that came across in the findings in Fig. 3 of this research in relation to the experiences of persons with online socialising technology. Technology enables harassment against marginalised gender groups on one hand and it blocks queer content uploads on the other hand. Technology assists the public to comment with full bigotry and with misogynistic and transphobic remarks on online applications with commenting capabilities, without blocking it. Yet, when say a transgender man attempts to upload content onto his personal YouTube channel, more than half of the videos are blocked from the category kid-friendly ‘restricted mode’. Transgender persons are prime targets for doxing and harassment. Technology enables hackers with malicious intent, to search for identifying data about persons by searching social media websites, public databases and manipulating people into revealing confidential information and then publish this information publicly on the internet. Transgender persons are also susceptible to harassment from trans-exclusionary radical feminists (TERFs) on the internet. It is evident from this research that gender marginalised groups, such as the transgender community in Ireland, fear engaging with online social media and communication technology for safety and self-protection reasons. Further to this, it is safer for transgender persons to verify the genuineness of a person face to face in a public setting rather than online, even though online socialising enables transgender people to express their gender identity. These findings also resonate with the notion that technology is cultural structured and considered a cisgender male arena where any other gender fears technology (Bray 2007). In addition to this and similar to the findings in Sect. 4.2, there is evidence in Fig. 3 suggesting that social media profiles should add an optional field for persons to fill in pronouns, in an effort to promote an awareness of non-binary persons, and by educating people it will help to put a stop to queer bashing.

## Conclusion

This paper contributes to the recent research (Hicks 2019) that digital data systems used for public services, are experienced by members of the LGBTQI + community as embodying restricted notions of a sex/gender binary. Furthermore, our evidence through the lived experiences of the LGBTQI + community, strongly suggests that gender data is sensitive and that some find it upsetting to provide this data where it is not clearly relevant to the service. Although this research was conducted as a case study in Ireland, these are universal issues. The findings in this research highlight that there can be a difference between the in person experience and the digital data system experience in accessing public services for LGBTQI + members. Through the experience of a transgender woman in this study, it is evident that there is cultural inclusion and understanding towards the LGBTQI + community from the staff, but not all of the time, and it is the digital data systems for which staff must use that are reinforcing the restrictive heteronormative gender binary assumptions long after legislation and society has moved on. These findings correlate with Hick’s (2019) research on the computerisation of the British governments social systems. The findings in this research also support Hick’s (2019) findings and the notion that today’s arena of big data analytics, machine learning, automation and other artificial intelligence technologies are built on top of legacy systems with deep rooted mandatory heteronormative gender binary data structures where people identifying outside of the binary sex/gender categories of male/man or female/woman are rejected and treated as an anomaly in digital society. This research also contributes to Ruberg and Ruelos’s (2020) study indicating that sexual orientation is not always a fixed identifier of a person. This research highlights that the sex and gender identities of people are not fixed, evident from the gender variant or gender fluid identities as well as transgender identities of participants in this research. In addition to this, this study presented a large sample of respondents indicating at the time a bisexual sexuality which also supports Ruberg and Ruelos’s (2020) research.

Women have been made invisible in the “data gap” where the lives and experiences of men are taken to represent all of humanity (Criado-Perez 2019). If data is based primarily on male lived experience, the logical conclusion is that “big data is corrupted by big silences, the truths you get are half-truths, at best. And often for women, they aren’t true at all” (Criado-Perez 2019, p 13). If women are disappeared in technological data, then LGBTQI + persons are even more marginalised and restricted by fixed, binary gender data which does not recognise multiple or changing gender identities. The findings of the research presented here contribute to the call for a more nuanced, flexible, and inclusive approach to gender in a wide array of technological spaces. Closing the gender gap (and—essentially—making it more inclusive to non-binary gender) is of benefit to all; when we exclude (at least) half of humanity from knowledge production processes we also lose their transformative insights and contributions to political, economic, and social life, and (Criado Perez 2019).

Gender identity goes beyond one of two sex/gender categories female/woman or male/man, where respondents reported that gender inclusiveness is lacking in many technological domains. There is limited evidence within this research to confirm that data collection systems and new technologies are reinforcing gender discrimination through the design and development of systems that marginalise and exclude genders other than the predefined societal binary sex/gender combination female/woman and male/man. This research contributes to the existing literature on digital data and gender and the experiences of the LGBTQI + community. The research lends itself to future work including the intersectional experiences with age, race and ethnicity within the LGBTQI + community and experiences with digital data. This research can also form a basis for future research on the LGBTQI + community and working in IT and technology sectors.

## Data Availability

Not applicable.
